# A comparative strategy for single-nucleus and single-cell transcriptomes confirms accuracy in predicted cell-type expression from nuclear RNA

**DOI:** 10.1038/s41598-017-04426-w

**Published:** 2017-07-20

**Authors:** Blue B. Lake, Simone Codeluppi, Yun C. Yung, Derek Gao, Jerold Chun, Peter V. Kharchenko, Sten Linnarsson, Kun Zhang

**Affiliations:** 1 Department of Bioengineering, University of California, San Diego, La Jolla, CA USA; 2 Department of Medical Biochemistry and Biophysics, Karolinska Institute, SE-17177, Stockholm, Sweden; 3 Department of Physiology and Pharmacology, Karolinska Institutet, SE-17177, Stockholm, Sweden; 4 Sanford Burnham Prebys Medical Discovery Institute, La Jolla, CA USA; 5 Department of Biomedical Informatics, Harvard Medical School, Boston, MA USA

## Abstract

Significant heterogeneities in gene expression among individual cells are typically interrogated using single whole cell approaches. However, tissues that have highly interconnected processes, such as in the brain, present unique challenges. Single-nucleus RNA sequencing (SNS) has emerged as an alternative method of assessing a cell’s transcriptome through the use of isolated nuclei. However, studies directly comparing expression data between nuclei and whole cells are lacking. Here, we have characterized nuclear and whole cell transcriptomes in mouse single neurons and provided a normalization strategy to reduce method-specific differences related to the length of genic regions. We confirmed a high concordance between nuclear and whole cell transcriptomes in the expression of cell type and metabolic modeling markers, but less so for a subset of genes associated with mitochondrial respiration. Therefore, our results indicate that single-nucleus transcriptome sequencing provides an effective means to profile cell type expression dynamics in previously inaccessible tissues.

## Introduction

Single-cell gene expression profiling can reveal unique cell types and states co-existing within a tissue^1–3^, where individual transcriptomes may be influenced not only by their cellular identity, but also their intercellular connectivity^4^ and possibly unique genomic content^5–8^. However, the need for viable intact single cells can pose a major hurdle for solid tissues and organs, and will preclude the use of postmortem human repositories. Genomic studies have circumvented this issue through use of isolated nuclei^5, 7–9^, thereby opening the door for development of a highly scalable SNS pipeline^10^. However, while nuclear transcriptomes can be representative of the whole cell^10–14^, differences in type and proportion of RNA between the cytosol and nucleus do exist^15, 16^, and have not been thoroughly examined. To address the potential differences in transcriptomic profiles from nuclear and matched whole cell RNA, we have generated RNA sequencing data from single neuronal nuclei isolated from the adult mouse somatosensory (S1) cortex for a direct comparison with data sets previously generated on S1 whole cells^2^, and provided a foundation for analyzing and interpreting SNS data.

## Results

**Figure 1:**
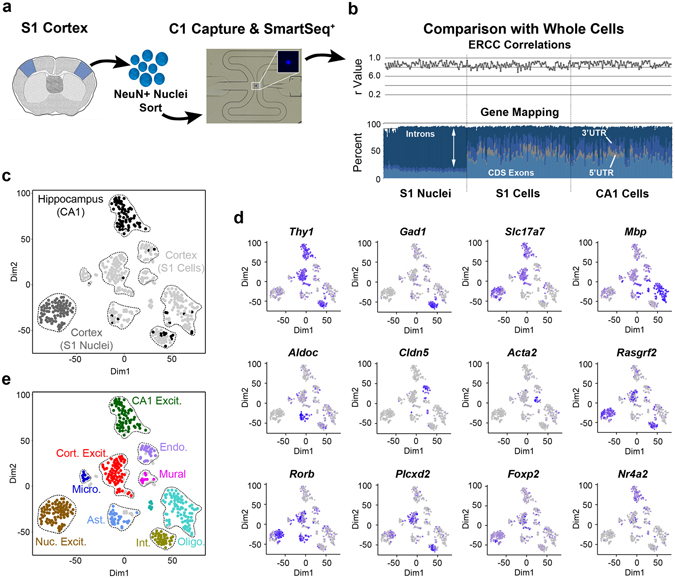
SNS reveals excitatory neuron identity. (**a**) Overview of the SNS pipeline. S1 mouse cortex was dissociated to single nuclei for NeuN+ and DAPI+ sorting and capture on C1 chips for modified SmartSeq (SmartSeq+) reactions. Inset shows DAPI positive nuclei in the C1 capture site. (**b**) Comparison of nuclear data sets with 100 random single S1 cortical or CA1 hippocampal data sets^2^. Top panel: Pearson correlation (r) coefficients for comparison of ERCC TPM values with their input quantities. Bottom panel: proportion of genomic reads mapping to coding sequences (CDS Exons), introns, or untranslated regions (3′ or 5′ UTRs). (**c**) t-SNE plots showing cluster distribution of hippocampal CA1, cortical S1 cells and cortical S1 nuclei. (**d**) t-SNE plots as in (**c**) showing positive expression levels (low – gray; high – blue) of cell type marker genes for oligodendrocytes (*Mbp*), astrocytes (*Aldoc*), endothelial cells (*Cldn5*), mural cells (*Acta2*), neurons (*Thy1*), inhibitory neurons (*Gad1*), excitatory neurons (*Slc17a7*), and excitatory neuron subtypes *Rasgrf2* (layer 2–3), *Rorb* (layer 4), *Plcxd2* (layer 5), *FoxP2* (layer 6) and *Nr4a2* (layer 6b)^2, 29^. (**e**) t-SNE plots showing expected identity of cluster groupings based on markers in (**d**) (Table S1, ambiguous data sets defined in Methods are shown in gray).

Single nuclei from frozen S1 cortex were isolated, flow sorted for neuronal nuclear antigen (NeuN) and processed for RNA-sequencing using a modified smart-seq protocol on the Fluidigm C1 system^10^ (Fig. 1a). Overall, nuclear and cellular data (Supplementary Table S1) showed similar numbers and types of genes detected (S1 nuclei - mean 5619 genes; S1 cells - mean 4797 genes; hippocampal CA1 cells - mean 6402 genes; Fig. 1b, Supplementary Fig. S1). ERCC spike-in RNA transcripts^17^ further confirmed high technical consistency (S1 nuclei - mean Pearson r = 0.86; S1 cells – mean r = 0.84; CA1 cells – mean r – 0.87; Fig. 1b, Supplementary Fig. S1). However, nuclear data sets showed a high proportion of reads mapping to intron regions (Fig. 1b), consistent with expected nascent transcripts present in the nucleus^18^. To ensure consistency between the different methodologies used to generate nuclear and cellular data, gene expression estimates were based on all genomic reads, including reads mapping to introns which have been found to accurately predict gene expression levels^10, 19^. Furthermore, inclusion of intronic reads ensured comparable read depth for nuclear data having low exon coverage (Fig. 1b).

**Figure 2:**
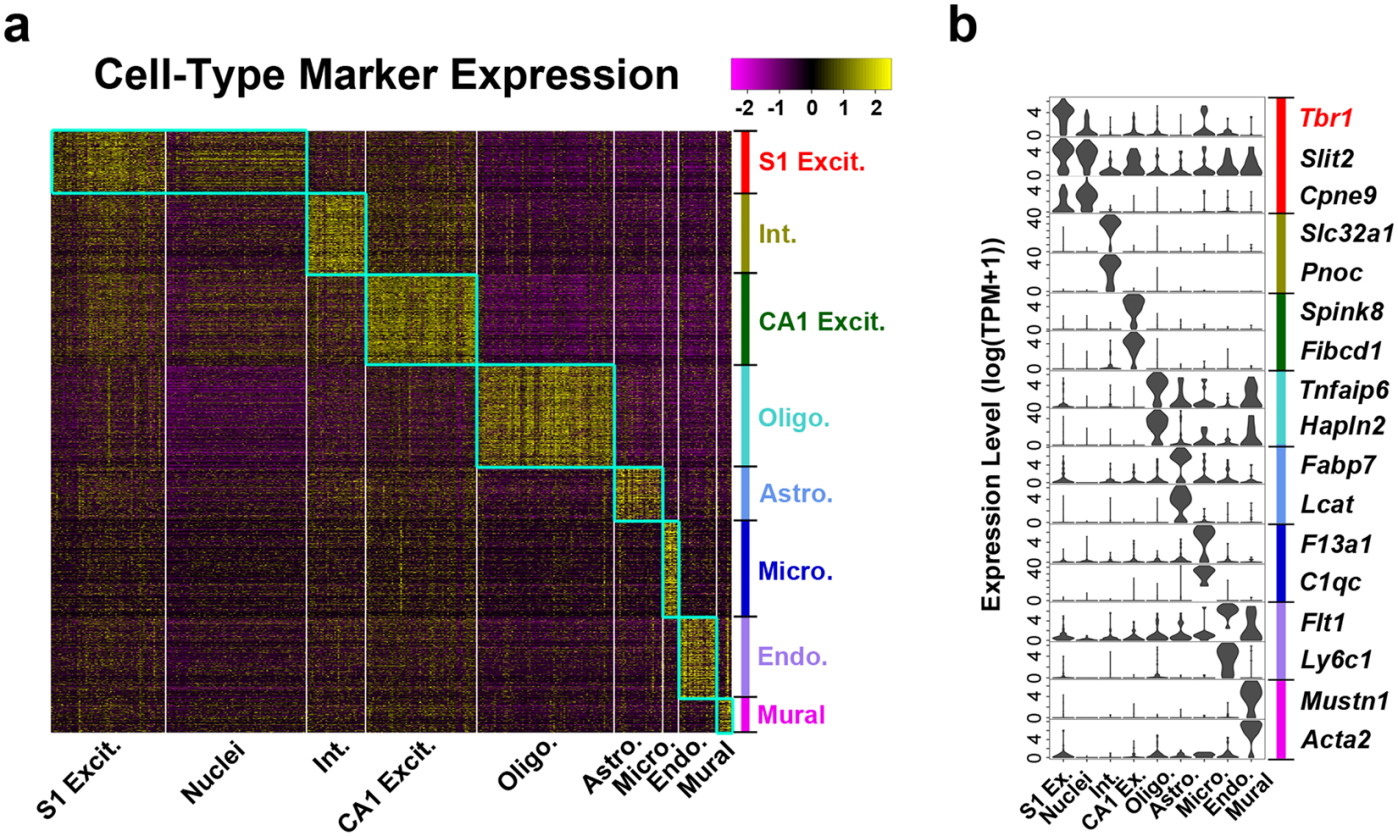
Nuclear transcriptomes accurately predict cell type. (**a**) Expression heatmap for cell type marker gene sets (colored bar) across all nuclear and cellular clusters (Fig. 1e). (**b**) Violin plots showing expression of select cell type marker genes across clusters.

To identify cellular identity, nuclear data sets were combined with randomly selected whole cell S1 cortical and CA1 hippocampal data sets^2^ for principal component analysis, dimension reduction through t-Distributed Stochastic Neighbor Embedding (t-SNE) and density clustering^1^ (Fig. 1c–e, Supplementary Fig. S1). Cellular clusters showed unique marker gene expression (Fig. 1d) that permitted cell-type classification^2^ (Fig. 1e). Neuronal nuclei, having low expression of the pan-neuronal marker *Thy1* (Fig. 1d) and clustering separately from cellular data (Fig. 1e), could still be classified as S1 cortical excitatory neurons based on expression of the excitatory neuronal marker *Slc17a7* and markers associated with upper layer cortical projection or granule neurons (Fig. 1d). The absence of inhibitory neuron data sets expressing *Gad1* from our NeuN sorted nuclei (Fig. 1d) likely reflects their expected lower abundance compared to excitatory neurons^10^ and their smaller nuclear size that may have been captured in limited fashion on the C1. In support of this presumption, nuclear expression of cell type-enriched genes^2^ (Supplementary Table S2) was consistent with S1 excitatory neurons, and not with other neuronal or non-neuronal cell types (Fig. 2a,b, Supplementary Fig. S2). Furthermore, the identified clusters were distinguished by biologically relevant genes, but not technical variability (Supplementary Fig. S1). Therefore, our results indicate that SNS accurately captures cellular identity.

**Figure 3:**
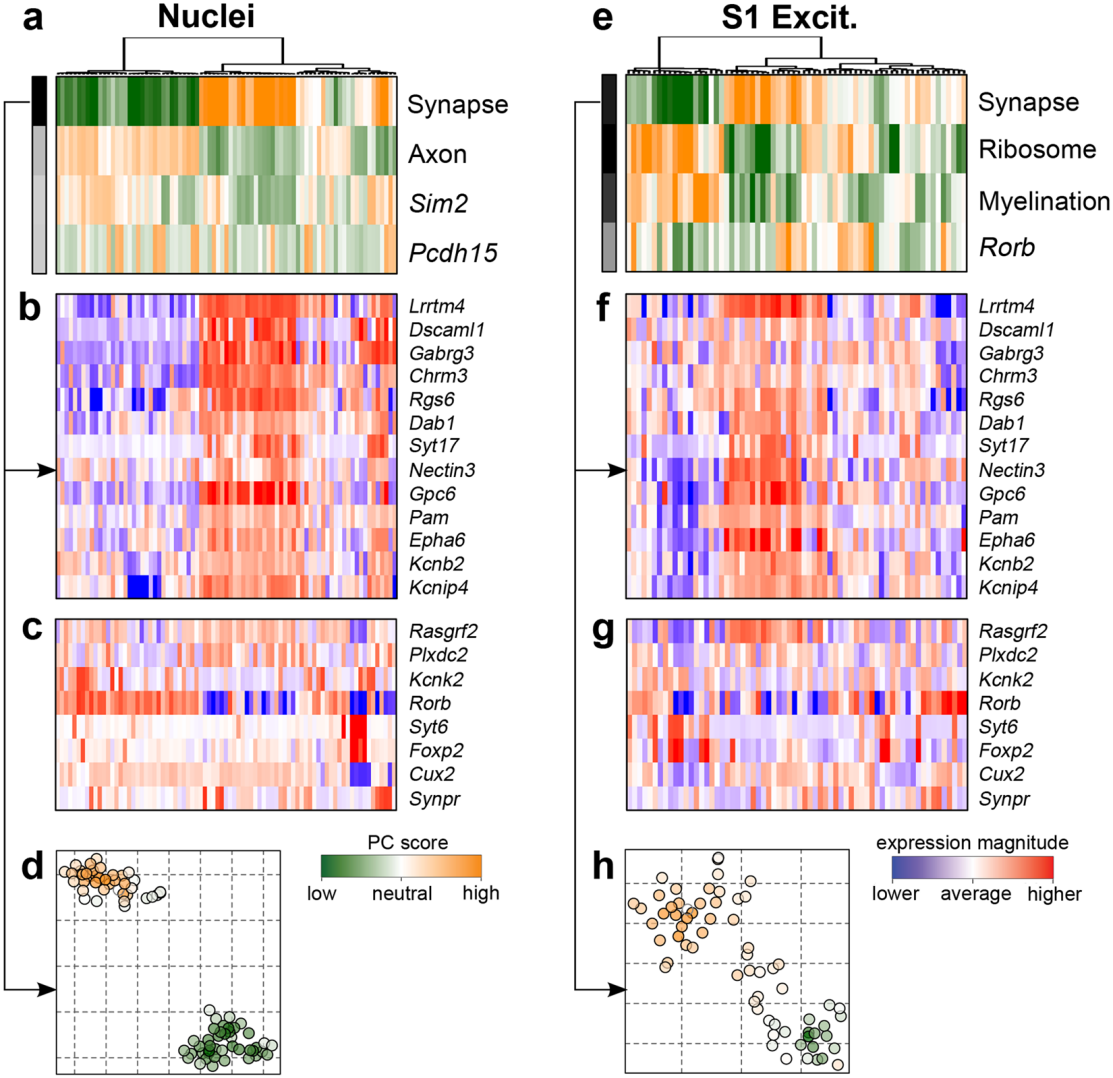
Transcriptional heterogeneity within the measured nuclei and corresponding whole-cell subpopulations. (**a**) Top four statistically significant aspects of heterogeneity (rows) are shown for the measured nuclei (columns), as identified by PAGODA^20^, labeled according to the key GO category or a gene driving each signature. (**b**) Expression patterns of genes driving the most prominent aspect, picked up by the synapse-associated GO category, are shown. (**c**) Expression of key marker genes defining subclasses of cortical neurons^2^ are shown. The synapse-distinguished neurons correspond to layer 2–3 (*Rasgrf2* ^+^) neurons. (**d**) A t-SNE embedding view, showing placement of the nuclei along the synapse-driven heterogeneity aspects shown in (**a**), which also separates two major subpopulations. (**e–h**) Analogous plots for an independent analysis of S1 excitatory whole cell neuron measurements. Expression of common synapse-associated (**b**) and marker (**c**) genes are shown (**f** and **g**) and t-SNE embedding (**h**) is driven by the synapse-associated aspect shown in (**e**).

To confirm that the single-nucleus data provides effective means to analyze cellular diversity, we have analyzed transcriptional heterogeneity within the measured nuclei, comparing it to the heterogeneity observed within the matched whole-cell measurements of the S1 excitatory neurons. Applying the PAGODA method^20^, we find statistically significant signatures distinguished within both nuclei and whole-cell measurements (Fig. 3a,e). The most prominent subpopulation within both measurements is driven by a large panel of synapse-associated genes, including *Lrrtm4* and *Gpc6* (Fig. 3b,f), and distinguishes neurons from layers 2–3 (*Rasgrf* ^+^) from the neurons originating from other layers, such as *Rorb* ^+^ layer 4–5, or *Foxp2* ^+^/*Syt6* ^+^ layer 6 neurons^2^, all of which are observed within both the measured nuclei and whole-cell populations, albeit at different proportions (Fig. 3c,g). Furthermore, these subpopulations show more distinct separation in nuclear data (Fig. 3d,h), which may underlie more defined type-specific expression observed from nuclear data compared with that of whole cells (Fig. 2a, Supplementary Fig. S2). Therefore, nuclear data accurately predicts cellular identity and provides an effective means for further subtype resolution.

**Figure 4:**
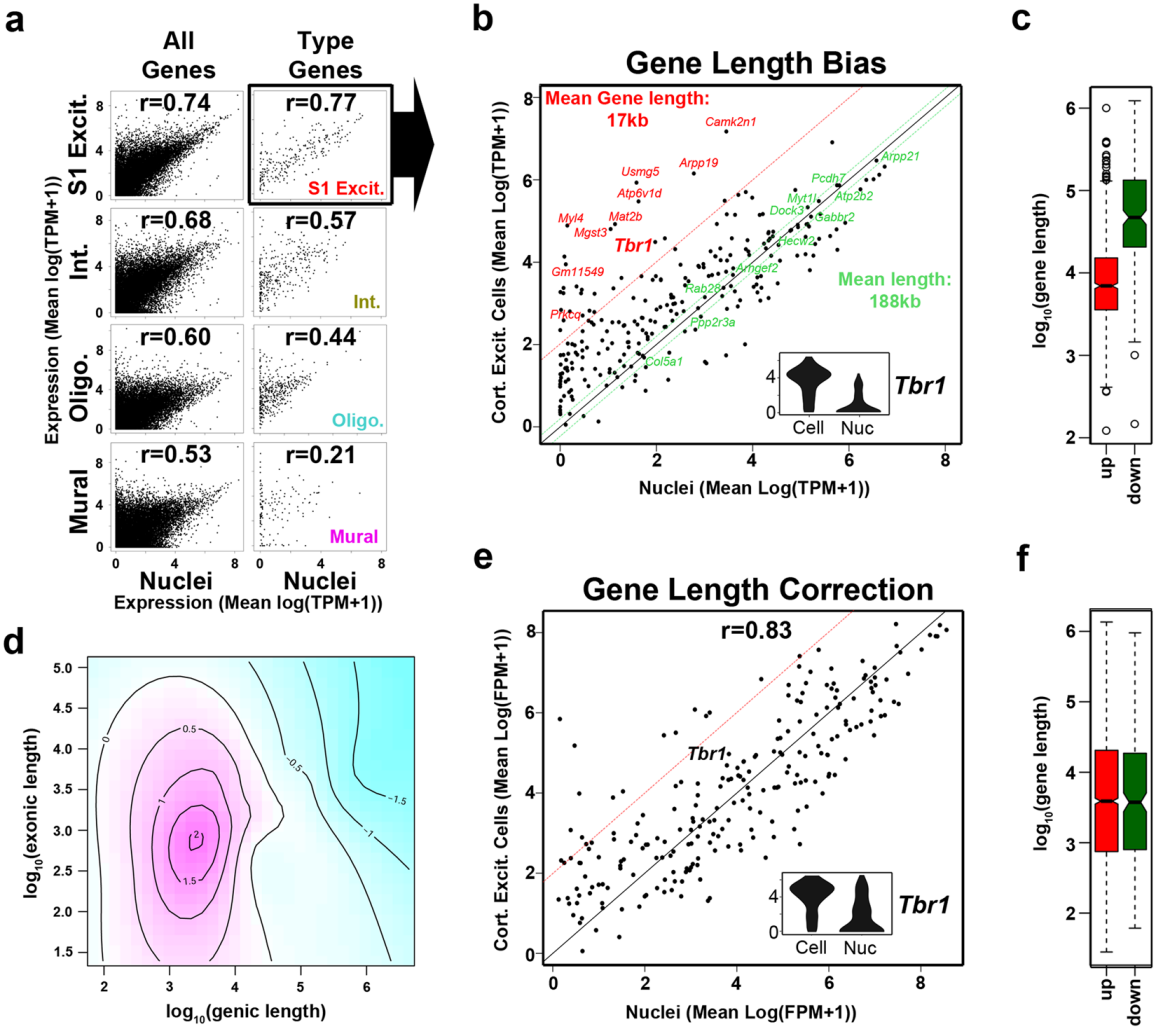
Gene length bias correction. (**a**) Scatter plots for nuclear and indicated cellular clusters using either all detected genes or the associated cell-type specific gene sets. Pearson correlation coefficients (r) are indicated. (**b**) Scatter plot indicated in (**a**) with genes detected higher in cells (red) or detected similarly between cells and nuclei (green) indicated. Inset is a violin plot of *Tbr1* expression. (**c**) Boxplot illustrating significant difference in average gene length between genes detected as up or down in cells over nuclei (Supplementary Fig. S4; Student t test, p = 6.41 × 10^−51^; Wilcoxon test, p = 3.77 × 10^−60^). (**d**) The systematic length bias in the whole cell – nucleus comparison was captured by the generalized additive model. The plot shows the interaction of total gene length (genic) and exonic length of a gene (pink – higher M values (log2 fold expression difference between whole cells and nuclei), blue – lower M values; the levels are labeled on the contours). (**e**) Scatter plot as shown in (**b**) after gene length correction showing improved *Tbr1* detection in nuclear data. (**f**) Boxplot on corrected expression values showing the absence of gene length bias (Supplementary Fig. S4; Student t test, p = 0.852; Wilcoxon test, p = 0.762).

However, nuclear RNA data, not surprisingly, did differ from that of whole cell RNA. For example, there was lower expression of the cortical pyramidal marker *Tbr1* (Fig. 2b), which shows robust expression in cortical projection neurons and plays an important role in their specification and functionality^21, 22^. Further, while averaged nuclear expression values showed the highest correlation with the S1 excitatory neurons (Fig. 4a, Supplementary Fig. S3), the degree of agreement varied depending on exonic gene length, or the total length of the genic region (Fig. 4b). Overall, genes that were better detected in whole cells over nuclei tended to be shorter, such as *Tbr1*, while genes showing better detection in the nuclei tended to be longer (Fig. 4c, Supplementary Fig. S4). This prominent length bias likely stems from the higher contribution of intronic reads in the nuclear measurements, as nascent RNAs of longer genes often include extensive intronic regions that would commonly be removed in the mature RNAs captured in the whole cells (Fig. 1b). We therefore developed a computational model to normalize systematic biases between whole cells and nuclei that were associated with gene length (genic) and intronic fraction (Fig. 4d). While the interaction of the gene length and intronic fraction explains only 17% of the observed expression variation, controlling for such dependencies allowed us to reduce length bias below statistically significant levels (Fig. 4e–f, Supplementary Fig. S4). The bias correction also partially recovered *Tbr1* expression in nuclei (Fig. 4e) and enabled more consistent overall expression of marker genes between matched nuclei and whole cells (Supplementary Fig. S4). Furthermore, application of this gene length bias correction to all data sets did not affect cell type classification (Supplementary Fig. S5). Therefore, we have developed an approach to normalize technical differences associated with differing maturity levels of transcripts detected between the nucleus and cytosol, while retaining biologically relevant gene expression dynamics.

**Figure 5:**
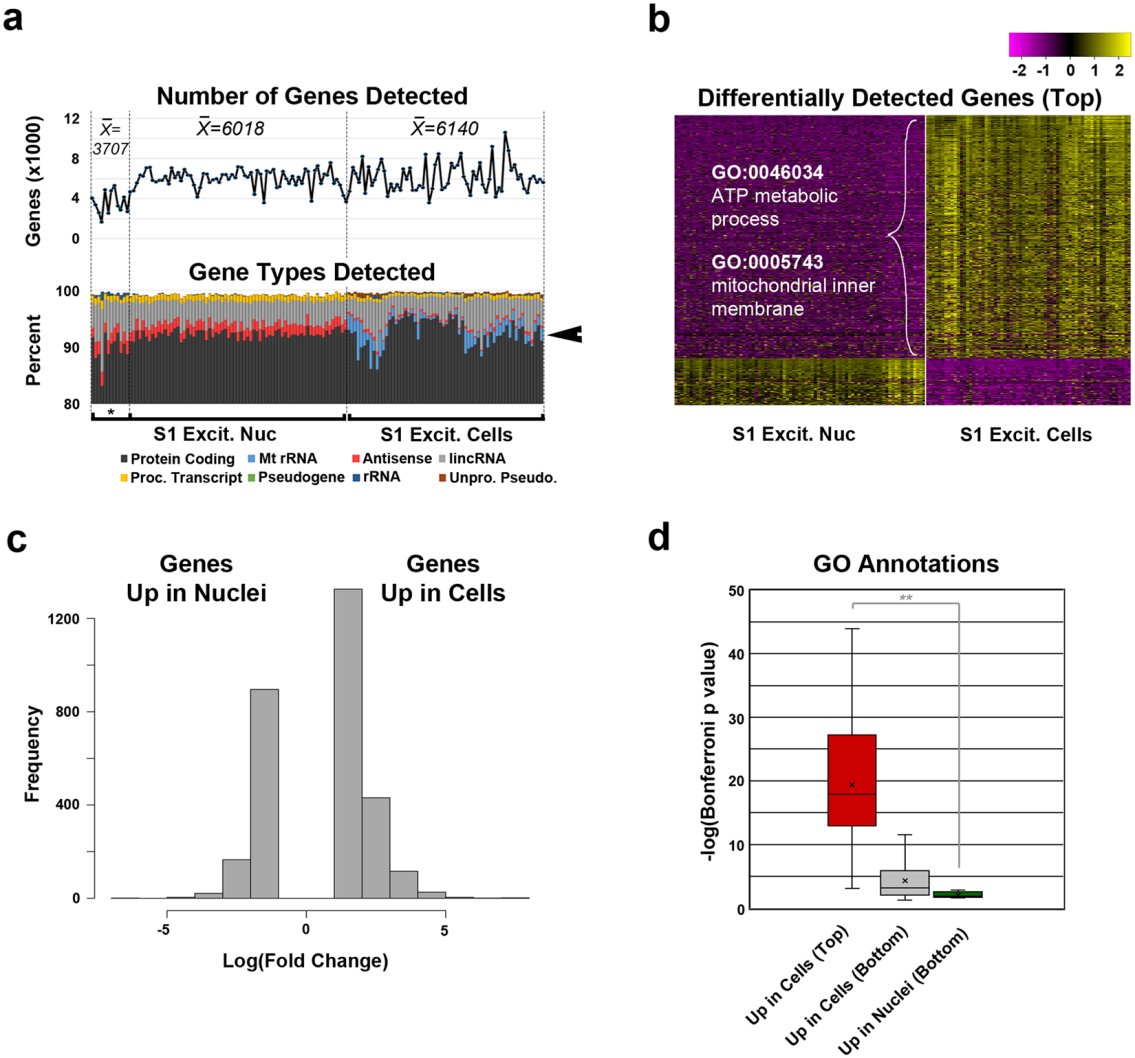
Differential transcript abundances between nuclei and whole cells. (**a**) Top panel: Total number of genes detected (count ≥ 4) from nuclei (*Indicates data sets generated from sorted nuclei frozen prior to C1 loading) and whole cell data sets representing S1 excitatory neurons. Lower panel: percentage of gene types detected, showing slightly more antisense transcripts detected in nuclear data and slightly more mitochondrial (Mt) rRNA detected in cellular data (arrow). (**b**) Heatmap of expression for top differentially detected genes (p < 1 × 10^−20^) between cellular and nuclear data sets showing representative GO annotations for genes over-represented in cells. (**c**) Histogram showing a higher frequency of genes that were better detected in cellular compared to nuclear data sets for S1 excitatory neurons (Supplementary Table S3). (**d**) Box plot showing significance values for annotations of top (p < 1 × 10^−20^) and bottom (p ≥ 1 × 10^−20^) differentially detected genes (Biological Process and Cellular Component categories, Supplementary Tables S4–S5). Student t-test p value is indicated: **p = 0.0002.

Application of this approach allows for good concordance between the nuclear and whole cellular transcriptome, yet additional sources of nucleocytoplasmic differences in transcript abundance may arise from mitochondrial transcription, splicing or nuclear export rates^18^, or post-transcriptional regulatory mechanisms^16^. To better understand the transcriptomic differences relevant to biological differences, we examined genes showing differential transcript accumulation between cell-type matched nuclei and whole cells using corrected expression data (Fig. 5, Supplementary Table S3). While only a slightly higher proportion of mitochondrial RNA was detected in cellular data (Fig. 5a), the majority of differentially abundant transcripts were cellular (Fig. 5b,c) and associated with respiratory and metabolic ontologies (Supplementary Table S4). Genes with transcript accumulation in nuclear over cellular data did show some functional ontologies (Supplementary Table S5), but these annotations had significantly lower p-values compared to those of cellular respiration (Fig. 5d). This likely reflects the more exclusive detection of respiratory-related transcripts in cellular data, compared to only an enrichment of neuronal-related transcripts in nuclear data (Supplementary Fig. S6). In fact, genes that did show more exclusive detection in nuclear data (Fig. 5b) failed to show these functional annotations (Supplementary Table S5). Therefore, our data confirms a high concordance in the nuclear and whole cell transcriptomes, with the main exception of cellular respiration transcripts accumulated in the cytosol.

## Discussion

Significant progress in understanding tissue heterogeneity has been achieved through large scale transcriptomic studies^1–3, 23^, providing extensive subtype composition of complex tissues and greater insight into their concerted functionality. However, many banked specimens at repositories – such as brain or tumor tissues – are not amendable to single-cell RNA sequencing methodologies due to a requirement for intact viable single cells. Furthermore, even for tissues that can be available directly from biopsy, the stress of whole cell dissociation may itself influence the expression of certain genes^24^. As such, we have developed a scalable SNS pipeline that can be applied to complex and difficult to study fresh or frozen material, and that permits extensive subtype resolution^10^. In order to address potential limitations of nuclei, the nuclear transcriptomes of mouse cortical excitatory neurons were compared with those derived from whole cells^2^. While SNS on mouse brain nuclei provided more limited cell type sampling compared to whole cells, newer methodologies continue to evolve to more comprehensively profile different cell types of a tissue using their nuclear transcriptomes^11^. Importantly for these studies, we provide evidence for accurate prediction of subtype-defining marker gene expression by nuclear transcriptome profiling of excitatory neurons (Figs 2 and 4a), which we expect to be generally applicable to all neuronal and non-neuronal cell types, as well as the ability to effectively resolve transcriptional subpopulations within a narrowly-defined cell type, identifying excitatory neurons originating from different layers (Fig. 3).

We find that the single nucleus and whole cell transcriptomes correlate highly, yet exhibit technical differences due to the natural abundance of nascent transcripts present in nuclei^18^ (Fig. 4). Since comprehensive understanding of gene expression dynamics in complex tissues will likely require intersection of data sets across multiple studies and modalities, we provide a normalization strategy that can reduce technical biases arising from comparisons of nuclear and cellular data (See Methods). Transcript abundance differences retained after normalization (Fig. 5b) likely represent true biological divergences. Consistently, while normalized data showed similar cell type resolution, nuclear and cellular data from cortical excitatory neurons continued to cluster separately (Supplementary Fig. S5). This likely reflects RNA composition differences found between nuclear and cytosolic compartments that, while not directly interrogated by this study, limit integrated analyses of cellular and nuclear data. Some enrichment of cell type-specific functional transcripts was observed in nuclei, however, this might in fact underlie different proportions of layer-specific excitatory neurons in nuclear and cellular data sets (Figs 1d and 3c,g), nascent transcription associated with early responses to neuronal activities^24^ or slight differences persisting in nuclear versus whole cell comparisons. By contrast, there was an almost exclusive detection of mitochondrial respiration-associated transcripts in whole cell data sets (Fig. 5). This may be attributed to the post-mitotic state of neurons, as neuronal progenitors instead accumulated transcripts associated with cellular division in their nuclei^12^. These findings highlight the potential for cell state-dependent transcriptomic differences that may arise between nuclear and cytosolic fractions.

We have demonstrated that SNS accurately captures expression of a majority of cell-type specific and functionally relevant genes in post-mitotic cells, while showing under-representation of certain transcripts related to cellular physiology that may be more rapidly exported from the nucleus^25^. Interestingly, the majority of genes associated with the genome-scale metabolic reconstruction (iMM1415) were accurately predicted from nuclear RNA (Supplementary Fig. S6), demonstrating the retained potential for *in silico* cell-type specific metabolic modeling from nuclear transcriptomic data^26, 27^. Therefore, single-nucleus transcriptomic sequencing provides an effective method for characterizing functionally relevant gene expression profiles and metabolic modeling of individual cells from tissues previously precluded from single-cell analyses.

## Methods

### Sample Origin and Nuclei Preparation

Animal handling and tissue harvesting methods were performed in accordance with the guidelines and regulations of the local animal protection legislation and were approved by the local committee for ethical experiments on laboratory animals (Stockholms Norra Djurförsöksetiska nämnd, Sweden). Postnatal day 21 wild-type CD1 mice of both sexes were perfused with cold and oxygenated artificial cerebrospinal fluid solution. The brains were then harvested and the somatosensory cortex isolated by dissection, snap frozen and stored at −80 °C until used. Neuronal nuclei were prepared using nuclear extraction buffer for nuclei isolation, stained with the neuronal nuclear antigen marker NeuN and flow sorted using single cell purity mode on a Beckman Coulter MoFlo Astrios EQ as described previously^10^.

### Nuclei Loading, RNA-Seq Library Preparation and Sequencing

For use on the Fluidigm C1 Single-Cell Auto Prep Array for mRNA Seq (Fluidigm, Cat# 100–5761), nuclei were either used directly after sorting or thawed rapidly from a DMSO frozen stock stored at −80 °C. Nuclei were loaded at ~120 nuclei/µl (5–10 µm capture sites, small chip) and RNA-seq libraries generated using a modified SmartSeq protocol containing both a supplemental random primer and PolydIdC as described previously^10^. For single nucleus libraries, 5 µL of cDNA were transferred to 96-well plates (Biorad, Cat# 9601) and normalized to 0.2 ng/µL in water using the EpiMotion (Eppendorf) liquid handling robot. Sequencing library preparation was performed as per the Fluidigm protocol. Libraries were subsequently sequenced on a HiSeq 2500 instrument (Illumina), using 50 bp single-end sequencing with dual index reads (2 × 8 bp). Raw sequence Fastq files were generated after sequencing runs using the BaseSpace Fastq generation algorithm (Illumina).

### RNA-seq data processing and analyses

Cellular data sets associated with the S1 cortex or CA1 hippocampus (Supplementary Table S1) were randomly selected for download from the GEO database. Single cell or nuclear reads were aligned to the mouse reference genome (GRCm38) using STAR (2.3.0) and assembled and quantified by HTSeq (v0.6.1) using Gencode vM8 annotations. ERCC spike-ins were mapped and quantified at the same time. Gene counts were converted to transcripts per million mapped reads (TPM) and log(TPM+1) was calculated. For ERCC TPM, calculations were based on ERCC counts only. Cells or nuclei with fewer than 1000 genes showing log(TPM + 1) of at least 1 were excluded. Genes that were expressed in less than 3 cells were excluded. Identification of cell type clusters, violin plots, scatter plots, and differential expression analysis were performed using Seurat software^1^ in R (code and data available at: *genome-tech.ucsd.edu/public/sNucSeqNorm*). To identify cell types, principal component analysis (PCA) was first performed on variable genes identified across single nucleus/cell data sets, then expanded to include all genes through PCA projection. tSNE and spectral density clustering (Seurat version 1.2) was used define clusters, with distance metrics based on the first 10 principal components determined to have significant p values based on a jack straw method. Outlier cells that failed to cluster (n = 12) or were considered to ambiguously cluster, having previously ascribed annotations^2^ that were contrary to the current cluster’s identity (n = 36; mostly oligodendrocytes, see Supplementary Table S1) and which showed marker gene expression associated with more than one cell type (Fig. 1d), were removed as a precautionary measure to exclude potential multiplets that were subsequently found to exist in this data set and which had these attributes^20^. Differentially detected genes between S1 excitatory cells and nuclei were identified using the “FindAllMarkers” function (Seurat version 1.4), using the t-test method and detection thresholds of log-fold change greater than 1.0 and p value less than 0.01. Heatmap for cell or nuclear predicted expression was generated for genes having p values less than 1 × 10^−20^. GO analyses were performed using the ToppFun function of the ToppGene suite (toppgene.cchmc.org) using default settings and with significance cutoff set at a Bonferroni adjusted p value of 0.05 and a maximum of 50 annotations per category.

### Gene Length Bias Correction

To correct for length bias in comparisons of nuclei and whole-cell measurements, the nuclear gene expression levels were generated using featureCount^28^ (FPM values) and were normalized by the expected expression magnitude, as estimated by a generalized additive model. Both HTSeq and featureCount methods for gene counting were tested and featureCount was selected based on the highest r correlation value of normalized nuclear and cellular data (r = 0.83 versus r = 0.82). The generalized additive model was built using mgcv R package, using smoothed term to model interaction of the total genic length and exonic length for each gene (on log10 scale), using Gaussian distribution with identity link: gam(M ~ s(t, e), family = gaussian(link = identity) where *M* is the log2 fold expression ratio between the nuclei and the whole-cell estimates, *t* is the total (genic) gene length, and *e* is the exonic gene length. Software and associated data are available at: *genome-tech.ucsd.edu/public/sNucSeqNorm*.

## Electronic supplementary material

Supplementary Information

Dataset 1
