# Supplementary material for: A comparative strategy for single-nucleus and single-cell transcriptomes confirms accuracy in predicted cell-type expression from nuclear RNA
Source: Sci Rep. 2017 Jul 20;7:6031. doi: 10.1038/s41598-017-04426-w (PMC5519641; doi:10.1038/s41598-017-04426-w)
Supplement: Supplementary file 1 — Supplementary Information [file 41598_2017_4426_MOESM1_ESM.pdf]

Title: A comparative strategy for single-nucleus and single-cell transcriptomes confirms accuracy in predicted cell-type expression from nuclear RNA.

Authors: Blue B. Lake[1]†, Simone Codeluppi[2,3]†, Yun C. Yung[4]†, Derek Gao[1], Jerold Chun[4]*, Peter V. Kharchenko[5]*, Sten Linnarsson[2]*, and Kun Zhang[1]*

Supplementary Material:

- Supplementary Tables S1-S5 (Excel file, .xlsx)
  - **Table S1.** Single cell or nucleus data sets used for analyses.
  - **Table S2.** Cell type-specific gene sets.
  - **Table S3.** Genes identified as differentially detected between S1 excitatory neuronal nuclei or cells.
  - **Table S4.** Go annotations for all genes over-represented in cellular over nuclear data sets for S1 excitatory neurons.
  - **Table S5.** Go annotations for all genes over-represented in nuclear over cellular data sets for S1 excitatory neurons
- Supplementary Figures S1-S6 (.pdf file)
  - **Fig. S1.** SNS is comparable to whole cell RNA-seq. **a.** Top panel: proportion of reads mapped to different gene types. Middle panel: relative proportion of all reads that were unmapped, multiply mapped or uniquely mapped to ERCC transcripts and reference genes. Bottom panel: the proportion of reads that mapped to either genome or ERCC. Results are shown for S1 nuclei, S1 cortical cells and CA1 hippocampal cells. **b.** Plots showing the frequency distribution of total number of reads sequenced (counts ≥4) and ERCC Pearson correlation r values [log (counts+0.1) versus log(concentration)] for all single nuclei libraries. **c.** Sample pairwise correlation plots using expression values (log(TPM+1)) from the top 20 genes from each significant PC loading used to cluster and classify cells in Fig. 1e. Associated sample clusters are indicated. **d.** Sample pairwise correlation plots based on ERCC TPM values (log(TPM+1)) with cluster identities indicated in (**c**). Hierarchical clustering was based on the "complete" method and euclidian distance.

- **Fig. S2.** Samples cluster based on cell type expression. **a.** Sample pairwise correlation plots using cell type-specific gene sets (log(TPM+1)) as shown in Fig. 2a (Supplementary Table S1-S2). Associated sample clusters are indicated. **b.** Hierarchical clustering of samples (based on "ward.D" method and euclidian distance) for heatmap shown in Fig. 2a, with cluster identities indicated in (**a**), and showing co-clustering of neuronal data sets (nuclear and cellular).
- **Fig. S3.** Nuclear data sets represent S1 excitatory neurons. Scatter plots showing averaged expression values for nuclei compared the indicated cell types. Left column shows plots using all genes, right column shows plots using only genes associated with the cell type being compared (Supplementary Table S2). Pearson correlation values (r) are indicated.
- **Fig. S4.** Correction of systematic gene length and intronic fraction bias. **a.** Pronounced length bias in comparison of whole cell and nuclei expression estimates. The scatter plot shows mean expression ($\log_{10}$ FPM) levels calculated across the measured nuclei (x-axis) and cells. The genes with mean log2 expression fold ratio (M value) over 3 are shown in red (higher in whole cells) and green (higher in nuclei). **b.** Correction based on the derived model (Fig. 4d) eliminates systematic length bias between whole-cell and nuclei estimates. The plot shows analysis of corrected values in the same way as shown in (**a**). **c.** Heatmap of S1 excitatory neuronal marker gene expression showing improved consistency across single cell/nuclei data sets after gene length bias correction.
- **Fig. S5.** Retained cell type classification after gene length bias correction. **a.** t-SNE plots showing expected identity of cluster groupings using corrected expression data and based on markers used in Fig. 1d. **b.** Heatmap of expression for clusters identified in (**a**) using cell type enriched marker genes Supplementary Table S2).
- **Fig. S6.** Differentially detected transcripts are more exclusively detected in cellular data. **a.** Heatmap of expression (log (TPM+1)) for top ($p < 1x10^{-20}$) differentially detected genes between nuclei and whole cells. **b.** Heatmap of expression (log (TPM+1)) for bottom ($p \geq 1x10^{-20}$) differentially detected genes between nuclei and whole cells. **c.** Scatter plot for nuclear and cellular data using using genes associated with the mouse genome-scale metabolic reconstruction (iMM1415, 1088 of 1415 genes detected). Pearson correlation

coefficient (r) is indicated. **d.** Heatmap of expression (log (TPM+1)) for detected iMM1415 genes.

Fig. S1

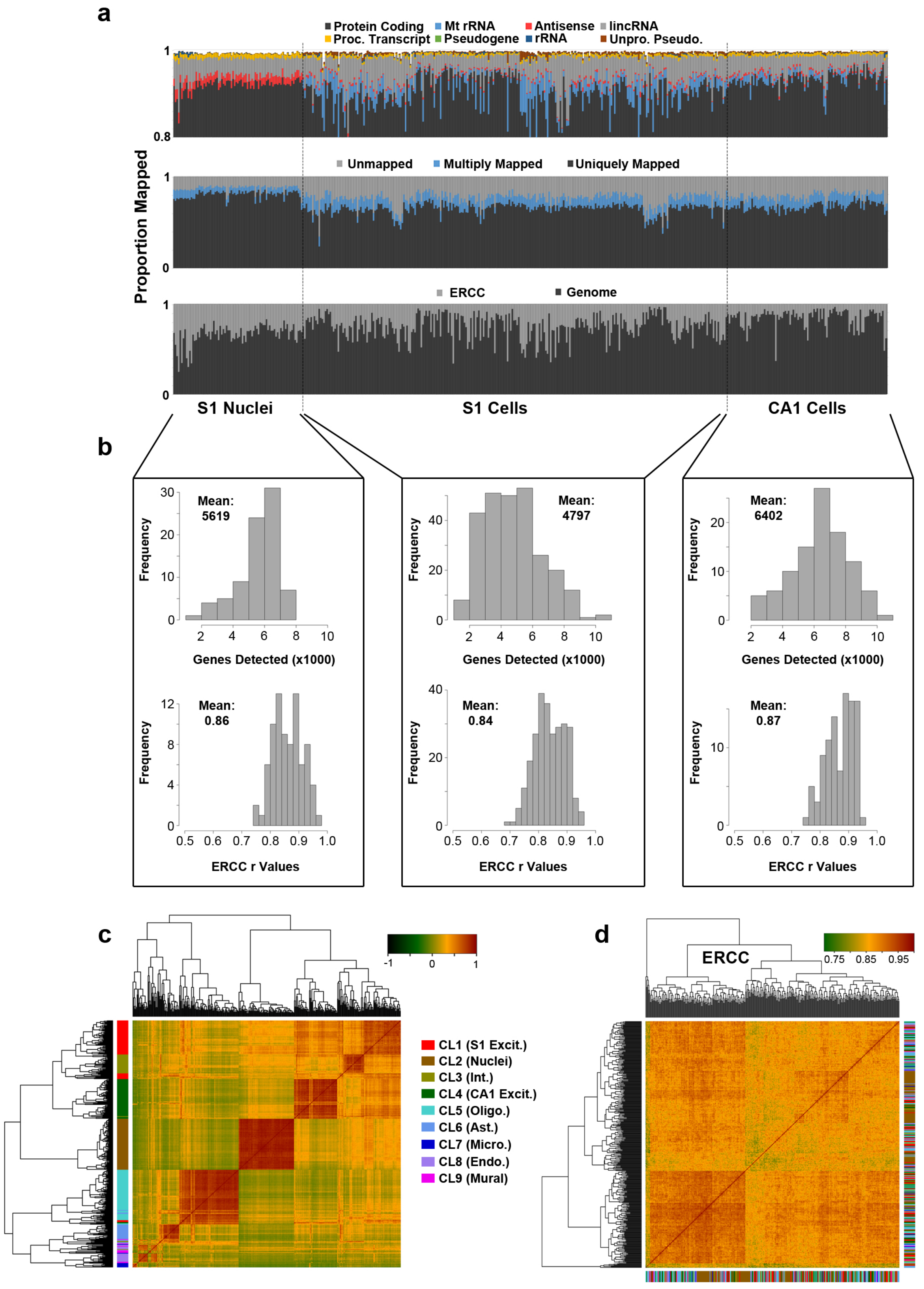
a
Protein Coding
Mt rRNA
Antisense
lincRNA
Proc. Transcript
Pseudogene
rRNA
Unpro. Pseudo.
Proportion Mapped
Unmapped
Multiply Mapped
Uniquely Mapped
ERCC
Genome
S1 Nuclei
S1 Cells
CA1 Cells
b
Frequency
Mean: 5619
Mean: 4797
Mean: 6402
Genes Detected (x1000)
Mean: 0.86
Mean: 0.84
Mean: 0.87
ERCC r Values
c
CL1 (S1 Excit.)
CL2 (Nuclei)
CL3 (Int.)
CL4 (CA1 Excit.)
CL5 (Oligo.)
CL6 (Ast.)
CL7 (Micro.)
CL8 (Endo.)
CL9 (Mural)
d
ERCC

Fig. S2

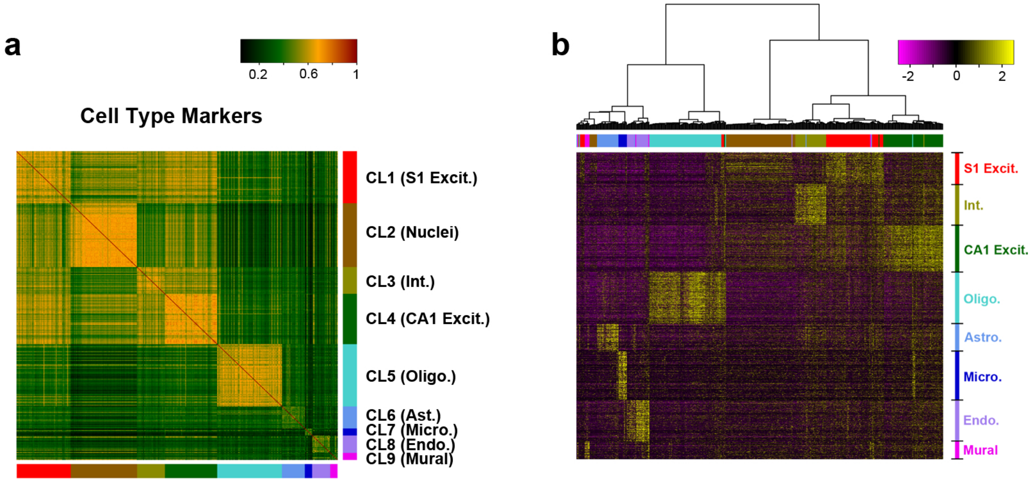
a
0.2
0.6
1
Cell Type Markers
CL1 (S1 Excit.)
CL2 (Nuclei)
CL3 (Int.)
CL4 (CA1 Excit.)
CL5 (Oligo.)
CL6 (Ast.)
CL7 (Micro.)
CL8 (Endo.)
CL9 (Mural)
b
-2
0
2
S1 Excit.
Int.
CA1 Excit.
Oligo.
Astro.
Micro.
Endo.
Mural

Fig. S3

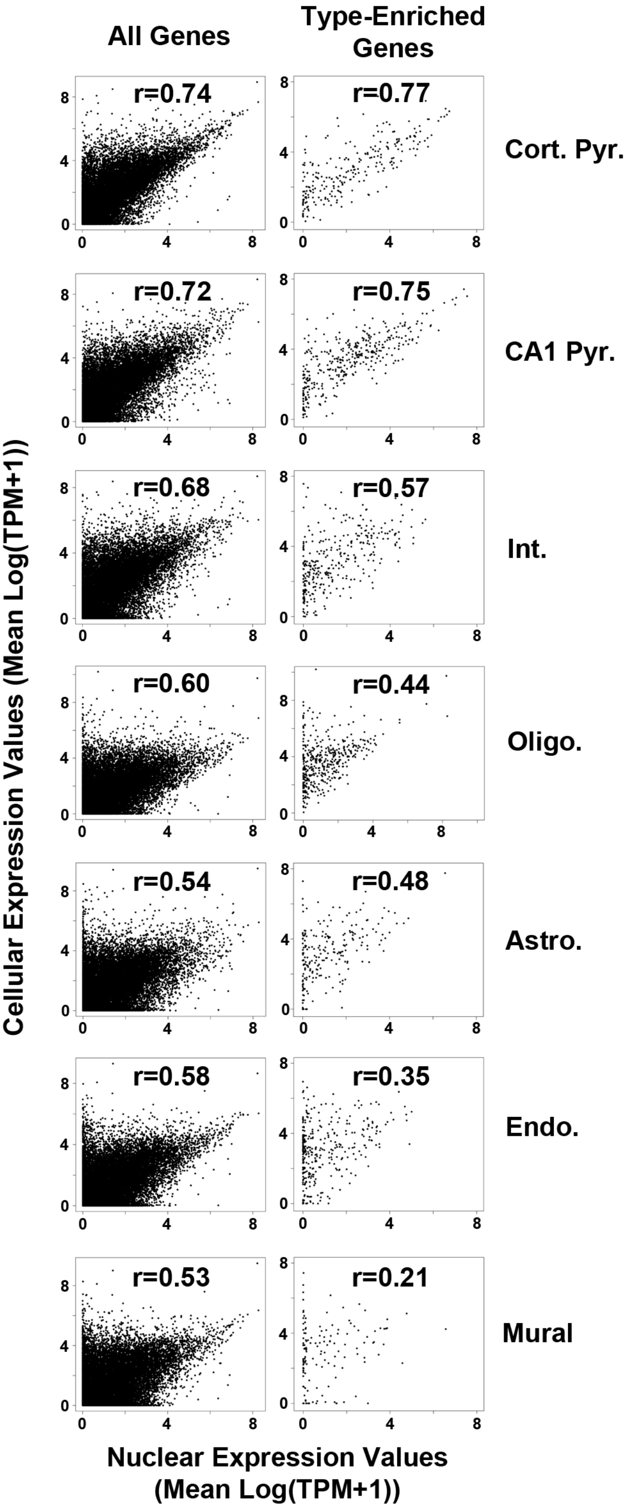
All Genes
Type-Enriched Genes
r=0.74
r=0.77
Cort. Pyr.
r=0.72
r=0.75
CA1 Pyr.
r=0.68
r=0.57
Int.
r=0.60
r=0.44
Oligo.
r=0.54
r=0.48
Astro.
r=0.58
r=0.35
Endo.
r=0.53
r=0.21
Mural
Cellular Expression Values (Mean Log(TPM+1))
Nuclear Expression Values (Mean Log(TPM+1))

Fig. S4

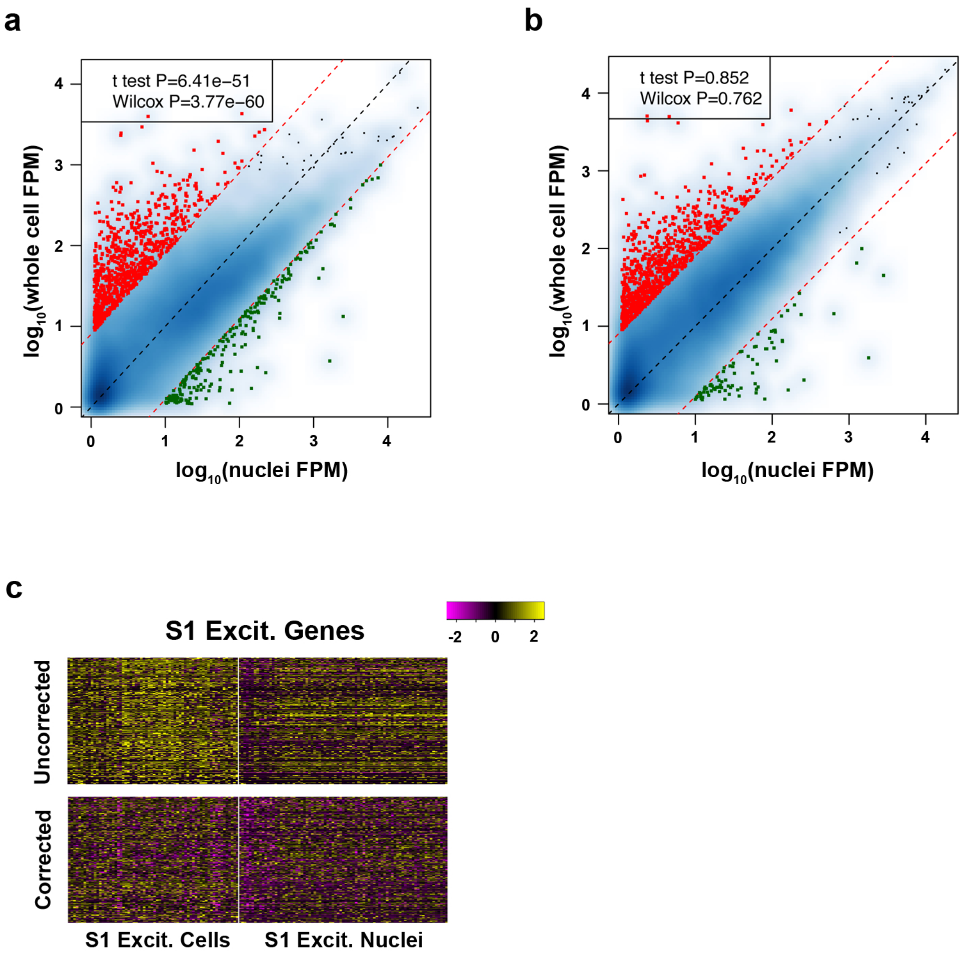
a
t test P=6.41e−51
Wilcox P=3.77e−60
log10(whole cell FPM)
log10(nuclei FPM)
b
t test P=0.852
Wilcox P=0.762
log10(whole cell FPM)
log10(nuclei FPM)
c
S1 Excit. Genes
-2
0
2
Uncorrected
Corrected
S1 Excit. Cells
S1 Excit. Nuclei

Fig. S5

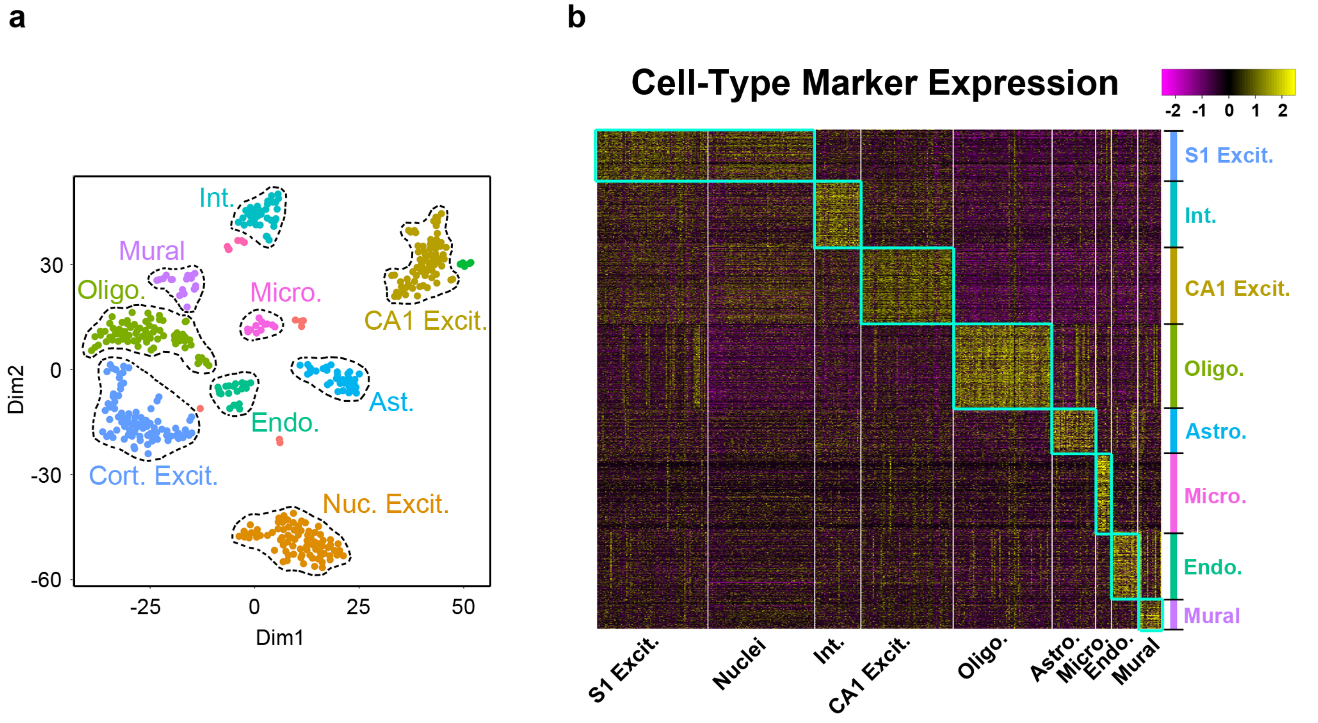
a
Int.
Mural
Oligo.
Micro.
CA1 Excit.
Ast.
Endo.
Cort. Excit.
Nuc. Excit.
30
0
-30
-60
-25
0
25
50
Dim1
Dim2
b
Cell-Type Marker Expression
-2
-1
0
1
2
S1 Excit.
Int.
CA1 Excit.
Oligo.
Astro.
Micro.
Endo.
Mural
S1 Excit.
Nuclei
Int.
CA1 Excit.
Oligo.
Astro.
Micro.
Endo.
Mural

Fig. S6

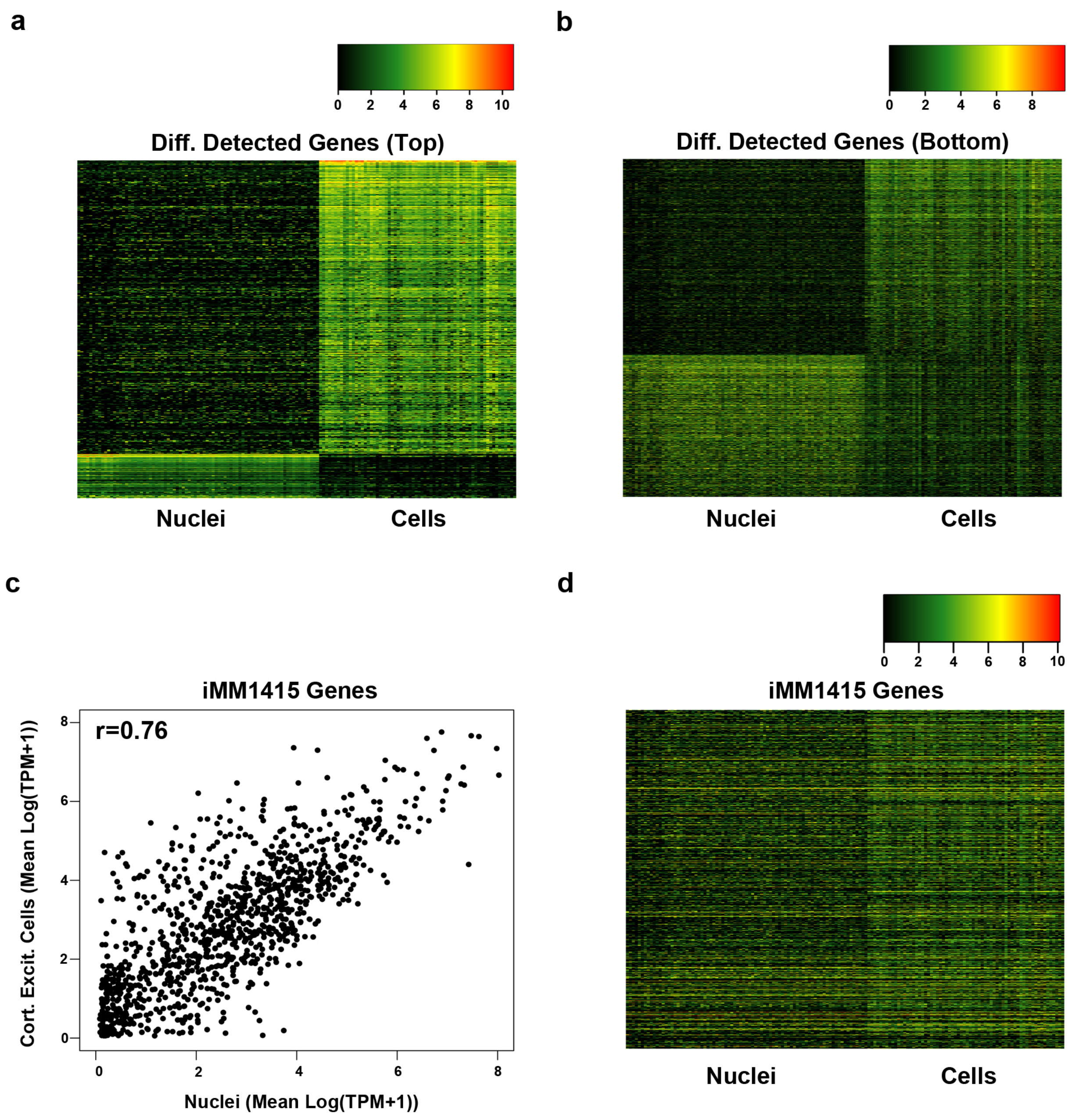
a
0
2
4
6
8
10
Diff. Detected Genes (Top)
Nuclei
Cells
b
0
2
4
6
8
Diff. Detected Genes (Bottom)
Nuclei
Cells
c
iMM1415 Genes
r=0.76
Cort. Excit. Cells (Mean Log(TPM+1))
Nuclei (Mean Log(TPM+1))
0
2
4
6
8
d
0
2
4
6
8
10
iMM1415 Genes
Nuclei
Cells
